# Engineered 3D vascular and neuronal networks in a microfluidic platform

**DOI:** 10.1038/s41598-018-23512-1

**Published:** 2018-03-26

**Authors:** Tatsuya Osaki, Vivek Sivathanu, Roger D. Kamm

**Affiliations:** 1 Department of Mechanical Engineering, Massachusetts institute of Technology, 77 Massachusetts Avenue, Cambridge, MA 02139 USA; 2 Department of Biological Engineering, Massachusetts institute of Technology, 77 Massachusetts Avenue, Cambridge, MA 02139 USA; 3 Singapore-MIT Alliance for Research & Technology, Singapore, Singapore

## Abstract

Neurovascular coupling plays a key role in the pathogenesis of neurodegenerative disorders including motor neuron disease (MND). *In vitro* models provide an opportunity to understand the pathogenesis of MND, and offer the potential for drug screening. Here, we describe a new 3D microvascular and neuronal network model in a microfluidic platform to investigate interactions between these two systems. Both 3D networks were established by co-culturing human embryonic stem (ES)-derived MN spheroids and endothelial cells (ECs) in microfluidic devices. Co-culture with ECs improves neurite elongation and neuronal connectivity as measured by Ca^2+^ oscillation. This improvement was regulated not only by paracrine signals such as brain-derived neurotrophic factor secreted by ECs but also through direct cell-cell interactions via the delta-notch pathway, promoting neuron differentiation and neuroprotection. Bi-directional signaling was observed in that the neural networks also affected vascular network formation under perfusion culture. This *in vitro* model could enable investigations of neuro-vascular coupling, essential to understanding the pathogenesis of neurodegenerative diseases including MNDs such as amyotrophic lateral sclerosis.

## Introduction

Motor neuron disease (MND) is a progressive neurological disorder accompanied by atrophy of the innervated muscle^1^ in which upper and/or lower MNs are damaged and become dysfunctional due to irreversible degeneration, resulting in loss of muscle strength. Although considerable effort has been devoted to understanding the mechanisms of MND, and various treatments established, the pathogenesis of MND is still unclear and could have multiple causes. Some of the main hypotheses are related to aging or genetic defects, abnormal function of glutamic acid (which causes MN death via overstimulation), abnormal accumulation of TDP-43 protein^2^, dysfunction of the redox properties of free radicals as well as dysfunction of neurovascular coupling including the depletion of neuronal growth factor released by the microvascular networks^3,4^.

Interaction between the neurons and vascular cells also plays an important role in regulating the neurovascular niche, controlling synaptic transmission and neuronal function^5^. For example, stabilization of neuronal circuits relies on matching metabolic demand and blood supply, as evidenced by the closely aligned anatomic arrangement of neural and capillary networks^6^. Vascular networks directly support neuron outgrowth and neuronal network development by efficient distribution of oxygen and nutrients^7^. Impairment of this support due to disruption of vascular flow or other types of vascular dysfunction is often the key to MN degeneration. For instance, in amyotrophic lateral sclerosis (ALS), the most common MND, and in the ALS mouse model (SOD1 mutation), blood-spinal cord barrier function of the EC monolayer in the spinal cord breaks down followed by vascular alterations such as insufficient nutrient delivery due to abnormal microvascular structures and leakage of blood components prior to MN degeneration and neurovascular inflammation induced by the loss of endothelial integrity. Furthermore, vascular endothelial growth factor (VEGF) secreted from the surrounding vasculature of neuron networks also influences the pathogenesis of ALS. ALS patients show low levels of VEGF expression early in the disease process, compromising neuro-protection^8,9^. Indeed, VEGF supplementation has been shown to be protective against ischemic MN death in an ALS mouse model^10^.

In spite of our understanding of the role of neurovascular interactions in the onset and progression of disease *in vivo*, effective treatment strategies and drugs have been elusive, in part due to the considerable gap between animal models and human disease^11,12^. Therefore, in order to understand the pathogenesis of MND in humans, we require an adequate *in vitro* model using human cells that mimics the *in vivo* interaction of vascular and neuronal networks.

Despite considerable effort to produce an *in vitro* neurovascular model, it is still not possible to adequately replicate *in vivo* neurovascular morphology. For instance, most established *in vitro* neuro-vascular models are 2D systems, utilizing a transwell-like approach to investigate either paracrine^13^ or juxtracrine^14^ cross-talk between neural cells and ECs. Notably, a partially 3D blood-brain barrier model was recently developed with a cylindrical microchannel in collagen gel^15^ to study neuro-inflammation with a co-culture of brain ECs, pericytes and astrocytes. Our group also reported a compartmentalized hydrogel-based co-culture microfluidic model with neurons, astrocytes, and brain ECs^16^. In neither system, however, do the ECs form *in vivo*-like microvascular networks, and the density of neural cells is much lower than found *in vivo*.

In other, non-neural settings, researchers independently demonstrated the formation of 3D vascular networks^17,18^ to study the angiogenic tendencies^19^ and extravasation of cancer cells^20^. Also, 2D and 3D dense neuronal networks^21–23^ have been grown in several types of hydrogel and on specific coatings such as poly-L-lysine (PLL), but these lack the vascular component. Despite these considerable advances, there remains no *in vitro* system that replicates the 3D morphology of intercalated neuronal and vascular networks with perfusion culture, which would be key to screening for compounds that influence the physiological interactions between these two systems.

Here, we engineered 3D neuronal networks and 3D perfusable vascular networks in both a macro-scale culture and microfluidic device using human ES-derived MNs and iPS-derived ECs (iPS-EC) networks. We demonstrate that microvascular networks promote synaptic connectivity via direct and indirect signaling under perfusion culture. Furthermore, MN networks were also found to influence vascular network formation. These results indicate that bi-directional signaling is critical to normal function and that our vascular and neuronal networks modeled in a microfluidic platform could promote a better understanding of their interactions in critical developmental processes, mechanobiological phenomena, and the pathogenesis of MND.

## Results

### Formation and characterization of MN spheroids

**Figure 1:**
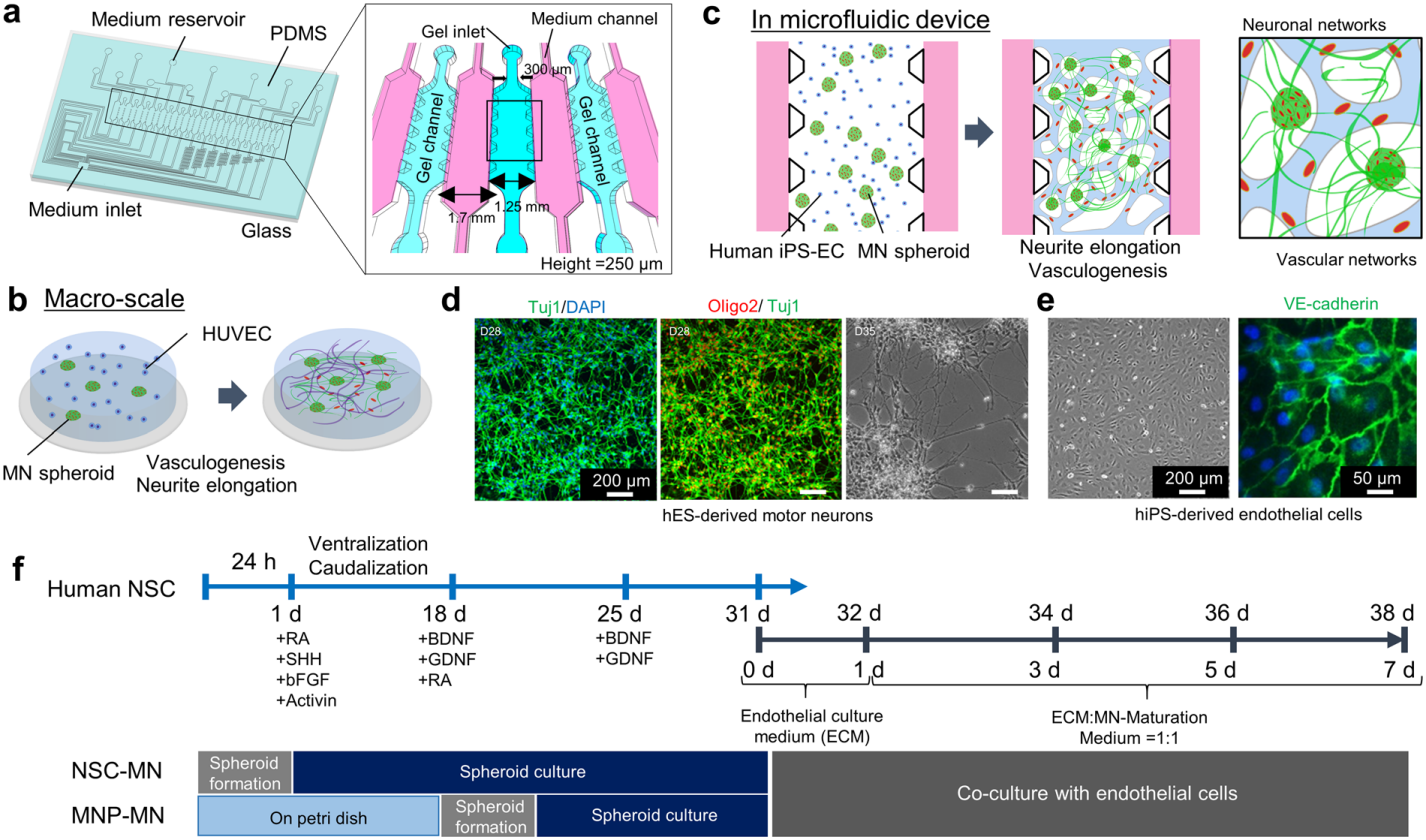
Schematic images of formation of neuronal and vascular networks. (**a**) The multi-channel microfluidic device consists of 14 independent gel channels separated by and adjacent to 15 medium channels. (**b**,**c**) Vascular and neuronal networks were formed in collagen gel on a petridish and in a microfluidic device by co-culturing motor neuron spheroids (MN spheroids) and endothelial cells (HUVEC, iPS-EC). (**d**) Characterization of differentiation into MN progenitor cells. Immunostaining of Tuj1 and Oligo2 showed that after 7 days of differentiation from neural stem cells, cells start to express Tuj1 which is an early neuronal marker, but not Oligo2 which is late MN progenitor marker. Mature MNs can be obtained by day 28. Phase contrast image shows the morphology of motor neurons on day 35. (**e**) Characterization of iPS-EC stained by VE-cadherin. (**f**) Time course of MN differentiation from human neural stem cells (ES-derived: H9). For formation of NSC derived MN spheroids (NSC-MN spheroids), NSCs were seeded into spheroid formation plate supplemented with NSC growth medium. Then, RA, SHH, bFGF, and Activin A were added on day 1 to induce differentiation. On day 18, BDNF and GDNF were added to promote maturation of MNs. For formation of progenitor-derived MN spheroids (MNP-MN spheroid), after NSCs were cultured in a Petri dish for 18 days, spheroids were formed. After ~30 days, MN spheroids and ECs were encapsulated in collagen gel within a microfluidic device and on Petri dish or the formation of MN and EC networks.

**Figure 2:**
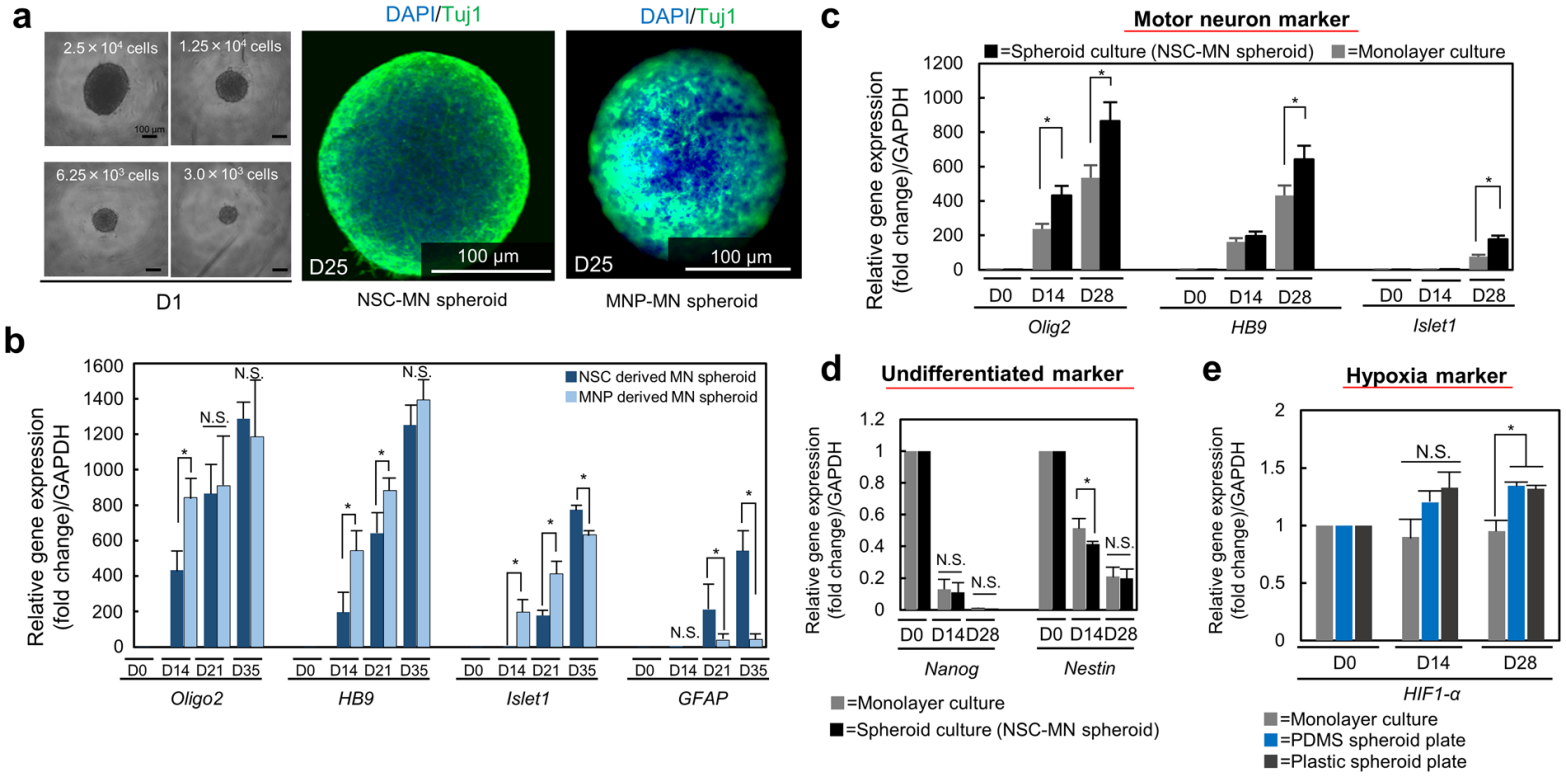
MN spheroid formation and characterization and morphogenesis of NSC-MN and MNP-MN spheroids. (**a**) The diameter of MN spheroids can be controlled by changing the seeding density. Immunostaining of Tuj1(green) and DAPI (blue) in NSC-MN and MNP spheroid. Left: NSC-MN spheroid. Right: MNP-MN spheroid. (**b**) Comparison of relative gene expression change of neuronal markers (*Oligo2*, *HB9*, *ISL1*, and *GFAP*) between NSC-MN spheroids and MNP-MN spheroids. NSC-MN spheroids have higher expression the gene related mature MN (*islet1*). In addition, the expression of *GFAP* which is astrocyte marker can be detected in NSC-MN spheroids. n = 4; *P < 0.01, two-way ANOVA. (**c**,**d**) Relative gene expression change of MN markers (*Oligo2*, *HB9*, *islet1*) and Nanog and neuronal stem cell marker (*Nestin*) showed that spheroid culture significantly improves differentiation into mature MNs compared to traditional monolayer culture. n = 5; *P < 0.01, two-way ANOVA. (**e**) No significant difference of hypoxia marker (*HIF-1α*) between the spheroid and monolayer culture. n = 3; *P < 0.01, two-way ANOVA, error bars ± SD.

It is well known that culturing MNs in neurospheres maintains suitable expression and characteristics (e.g. *Nestin* in the neural stem cell stage: *Tuj1*, *glial fibrillary acidic protein* (*GFAP)*, oligodendrocyte marker in the neural progenitor cell stage) long-term^24^. In this study, two different types of MN spheroids were formed to accelerate the differentiation from human ES-derived neuronal stem cells (hNSC) (Fig. 1f). Neural stem cell-derived MN (NSC-MN) spheroids and motor neuron progenitor cell-derived MN (MNP-MN) spheroids were formed using a sequential application of several growth factors (retinoic acid (RA), sonic hedgehog (SHH), bFGF, Activin A, brain-derived neurotrophic factors (BDNF) and glial cell-derived neurotrophic factor (GDNF)) as described in Materials and Methods. The differentiation protocol was optimized in monolayer culture (Fig. 1d). By changing the seeding density of cells in the spheroid formation plate (3.0 × 10^3^ cells/well to 2.5 × 10^4^ cells)^25^, spheroids with different diameter could be obtained (Fig. 2a). To prevent necrotic cell death at the core of the spheroid, spheroids with a diameter less than 200 μm were used.

To characterize and compare these two types of MN spheroids in terms of differentiation efficacy, mRNA was purified from both to perform RT-PCR analysis of several neuronal differentiation markers (*Oligo2*, *HB9*, and *islet1*, Fig. 2b). The expression of three MN markers increased with increasing culture time (Days 14, 28 and 35). In particular, the expression of islet1, a mature MN marker, was higher in NSC-MN spheroids than in the MNP-MN spheroids. In addition, increased expression of *GFAP*, a typical glial cell marker, could be observed in NSC-MN spheroids. This result indicates that NSC-MN spheroids are heterogeneous and likely contain not only of MNs but also glial cells such as astrocytes and Schwann cells.

Spheroid formation clearly improves the differentiation into MNs compared to monolayer culture (Fig. 2c). Even though spheroid and monolayer culture have comparable differentiation (caudalization and ventralization), maturation to MNs is superior in the spheroid culture judging from the expression of the undifferentiated markers *Nanog* and *Nestin* (Fig. 2d).

In addition, expression of the hypoxia marker (*HIF-1α*) did not increase significantly in spheroids compared to the monolayer culture (only 1.5X higher). Furthermore, no significant difference in gene expression of *HIF-1α* is seen between the PDMS and the plastic spheroid plates (Fig. 2e). This result suggests that the core of the neurosphere may not experience necrosis due to oxygen limitation, possibly due to the small diameter (<200 μm) (Fig. 2e)^26^. Other studies have shown that cell-cycle arrest occurs in spheroids greater than 300 μm in diameter due to hypoxia, and that necrotic cell death could be observed in spheroids larger than 400–500 μm in diameter^27,28^. High levels of HIF-1α expression are known to cause neuroinflammation. However, it has also been shown that HIF-1α improves neuronal survival during the early acute phase of ischemic stroke in the presence of ECs^29^. The activation of HIF-1α by MNs triggers secretion of VEGF through a paracrine response of the endothelium, leading to neuroprotection of MNs^30^.

**Figure 3:**
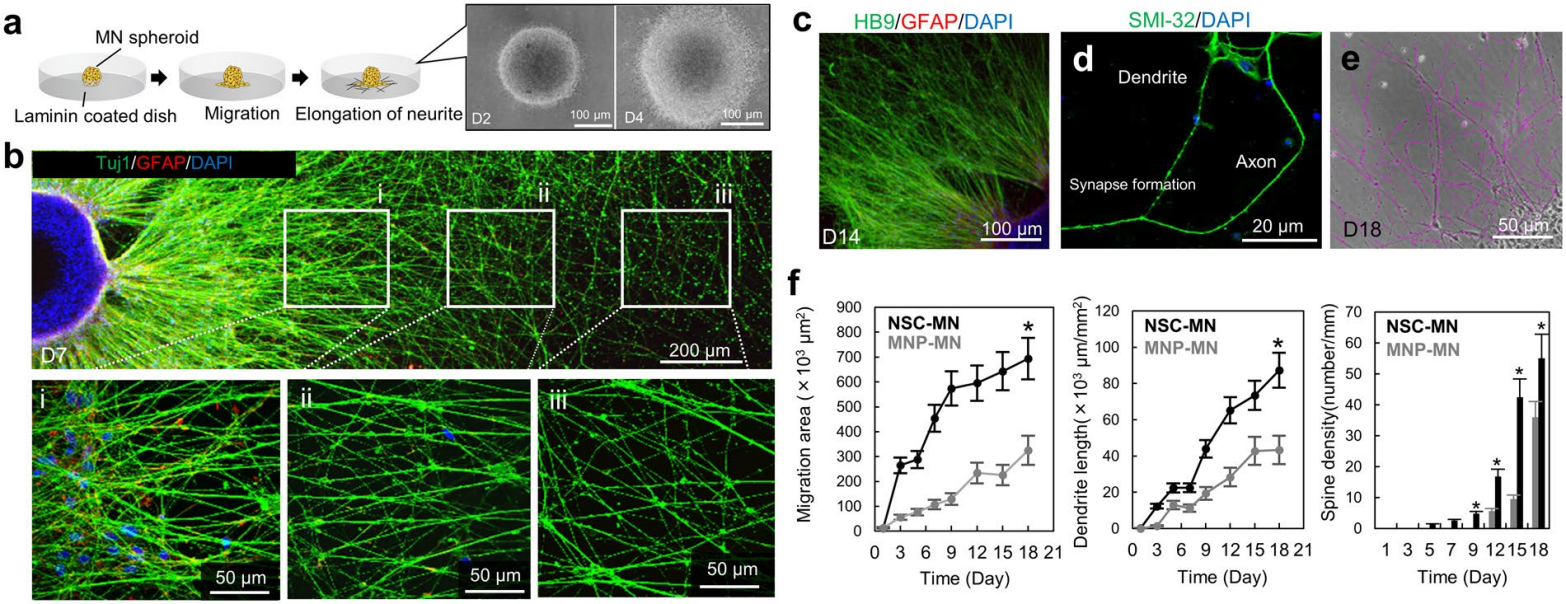
Morphogenesis of NSC-MN and MNP-MN spheroids. (**a**) To test the elongation of neurites, MN spheroids were reseeded on laminin-coated dishes and migration and elongation of MNs was observed and quantified. Representative images of morphological change of NSC-MN spheroids. (**b**) MN neurites from NSC-MN spheroids were visualized by immunostaining of HB9, GFAP, and DAPI at three different regions (i, ii and iii). Note that the regions marked in the zoomed-out view are representative. Both soma and neurites can be seen in (i), a few soma and neurites in (ii), and only neurites in (iii). (**c**) after 14 days of seeding, characterization stained by HB9, GFAP, and SMI-32 (**d**). (**e**) To calculate neurite properties, image processing is conducted. (**f**) Quantitative comparison between NSC-MN spheroids and MNP-MN spheroids in terms of migration area, neurite length and spine density. NSC-MN spheroids have a higher potential of migration, neurite elongation, and spine density. n = 3; *P < 0.01, one-way ANOVA between NSC-MN and MNP-MN. Error bars ± SD.

Differences in the morphology between NSC-MN and MNP-MN spheroids were evaluated by re-seeding MN spheroids on ornithine-laminin coated dishes after maturation (Fig. 3a). Both neurospheres attached to the laminin-coated surface and MNs started to migrate and disperse by around day 4 (Fig. 3b). We also observed neurite extensions from the neurospheroid on day 7 using HB9, a MN marker. This showed MN specific regular and dense spine structures and synapse formation of neurite (Fig. 3c,d). Image analysis showed significant differences between NSC-MN and MNP-MN in terms of the surface area covered by migrating and spreading cells, neurite length and spine density (Fig. 3e,f). Representative images at day 18 were shown in Supplementary Fig. S1. In particular, neurite length extending from NSC-MN spheroids was observed to be significantly greater than that of MNP-MN spheroids. It should be noted that spine density of NSC-MN spheroids, which regulates synaptic formation, connectivity, and activity, is also higher than in MNP-MN spheroids. These results are consistent with PCR analysis, which shows that NSC-MN spheroids consist of more mature MNs than the MNP-MN spheroid.

### Co-culture of MN spheroids and ECs in macro scale culture

**Figure 4:**
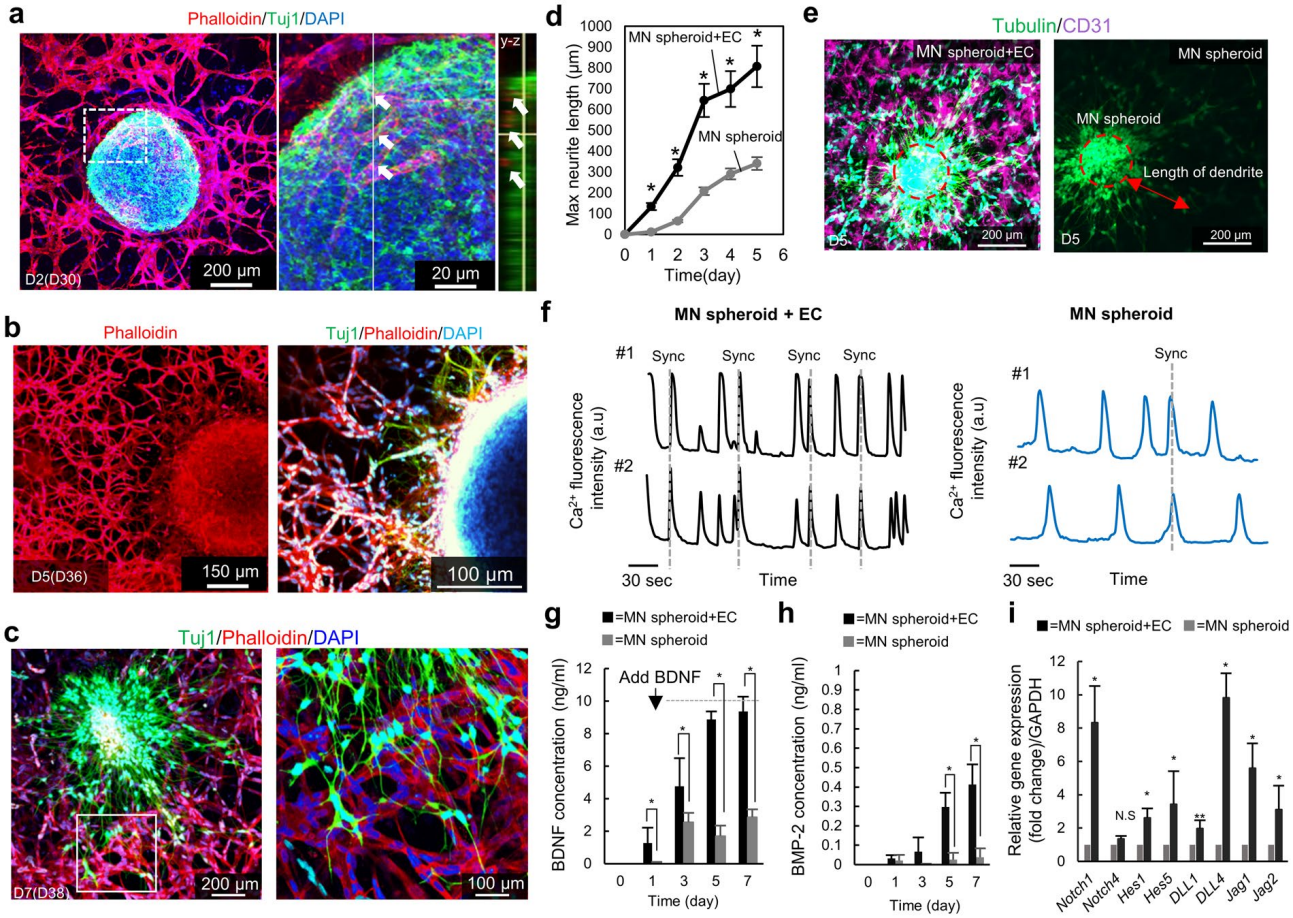
Co-culture of NSC-MN spheroids and HUVEC in collagen gel on a Petri dish and neuronal activity with and without HUVEC networks. (**a**) After 2 days of co-culture with HUVEC and MN spheroids, HUVEC formed well-connected microvascular networks around the spheroid. Almost all microvascular networks exist surrounding MN spheroids, but a few HUVECs invaded into the MN spheroids and formed networks inside. After 3, 4 days, MN spheroids start to migrate out of the initial spheroid and penetrate into the surrounding collagen gel, resulting in the formation of long neurites. (**b**) Immunostaining of actin, Tuj1 and DAPI show that vascular networks are formed prior to neurite elongation by Day 3. Neurite elongation is observed during the next several days. (**c**) After 7 days in culture, interpenetrating MN networks and vascular networks can be observed. MN neurites directly attach to the vascular networks enabling communication between them. (**d**) HUVEC networks promote neurite elongation of MNs. n = 3; *P < 0.01, two-way ANOVA. (**e**) Quantification of maximum neurite length. (**f**) Spontaneous Ca^2+^ oscillation by fluo-8am. EC networks increase the frequency, amplitude, and synchrony of neuron activity. (**g**,**h**) The concentration of BDNF and BMP2 in culture medium with and without HUVEC networks. HUVECs secreted BDNF which can upregulate neuronal differentiation and synapse formation and promote neuroprotection. In contrast, the concentration of BMP2 did not significantly increase to affect cellular activity. n = 3; *P < 0.01, two-way ANOVA. (**i**) Relative change of mRNA expression related to juxtacrine signaling. *Notch1* and *DLL4* are both significantly increased, resulting in synaptic stabilization. n = 3; *P < 0.01, two-way ANOVA. All error bars ± SD.

To initially test the interaction between MNs and ECs, NSC-MN spheroids and human umbilical vein endothelial cells (HUVECs) were encapsulated in a relatively large volume of rat tail collagen gel in a 35 mm diameter petri dish (“macro-scale culture”) (Fig. 1b). At the beginning of co-culture, the growth medium for ECs was used because the formation of vascular networks occurs rapidly via vasculogenesis. After 24 hours, the culture medium was replaced with a 1:1 mixture of endothelial culture medium and MN maturation medium to support neurite elongation. The initial process of vasculogenesis was key in forming well-connected networks that, once established, were found to be relatively stable. Within 3 days, well-connected vascular networks with *in vivo*-like morphology were formed around the neurosphere (Fig. 4a). During this initial phase, nearly all HUVECs formed into networks surrounding the MN spheroid, but some were also observed to invade into the MN clusters, forming microvascular networks inside the spheroid (Fig. 4a). After 3–4 days of co-culture, cells in the MN spheroid started to migrate and disperse into the surrounding matrix. Neurite elongation from the MNs in the neurosphere was visualized on day 5 by immunostaining of Tuj1 and F-actin (Fig. 4b). By day 7, neurites extended >800 μm from the neurosphere and several neurites and MN cell bodies directly attached to the vascular networks (Fig. 4c), perhaps to form specific junctions with N-cadherin^31^.

To evaluate the influence of ECs on MNs, maximum neurite length was compared with and without HUVEC networks in a collagen gel (Fig. 4d,e). The presence of vascular networks produces longer neurites, demonstrating that ECs improve neurite growth. In general, mature hippocampal neurons, cortical neurons, and MNs develop spontaneous oscillations in cytosolic Ca^2+^ concentration as a result of synapse formation in neuronal networks^32–35^. This oscillation and synchronous, rhythmic firing is a regulated function of neuronal networks^36^. We, therefore, introduced a fluorescent Ca^2+^ indicator (Fluo-8-AM) and subsequently observed spontaneous Ca^2+^ oscillations in a majority of the MNs. In addition, co-culture with ECs significantly upregulated Ca^2+^ signaling by the MNs as characterized by the frequency and amplitude of the signal, as well as synchronized spontaneous Ca^2+^ oscillations (Fig. 4f).

To study signaling between ECs and MNs, we measured the concentration of BMP-2 and BDNF, factors that are commonly associated with differentiation of neurons and necessary to maintain neuronal function (Fig. 4g,h). Although endothelial medium which was used at the beginning of co-culture for 24 hours does not contain BDNF, co-culture medium used after that contains 10 ng/ml of BDNF. Without HUVEC networks, the amount of BDNF decreased to ~2–3 ng/ml each day. On the other hand, the reduction of BDNF did not occur in the presence of HUVEC networks, resulting in maintenance of ~8–10 ng/ml of BDNF on days 5 and 7 (Fig. 4g). In contrast to BDNF, ECs secreted low, although statistically significant amounts of BMP-2 (<1 ng/ml) that would regulate neuronal differentiation (Fig. 4h). However, typical levels lie in the range of 1–100 ng/ml^37,38^. These results suggest that paracrine signaling via BDNF, but to a lesser extent BMP-2, secreted by ECs likely contributed to neurite elongation and activation of Ca^2+^ oscillations. BDNF is a key growth factor that accelerates neuronal differentiation^39^ by activation of *MAPK/ERK* and *PI3K/Akt* signaling pathways^40^. We, therefore, hypothesize that in co-culture with ECs, BDNF and potentially BMP-2 secreted by ECs and present in the culture medium upregulated neuronal differentiation and survival of neurons through autocrine signaling in co-culture system.

Analysis of gene expression showed that *Notch1*, *Delta-like 4 (Dll4)* (in ECs) and *Jag1* in co-culture of MN spheroids and ECs all significantly increased in comparison to only MN spheroids in collagen gel (Fig. 4i). In addition, *Hes1 and Hes5* expression, downstream of the delta-notch pathway in neurons, were also upregulated, resulting in inhibition of MN differentiation and stabilization of synapse formation. This result was consistent with previous findings^41,42^. As *Dll4* and *Jag1* are highly expressed by ECs^43^, this raises the possibility of *Dll4-Notch1* and *Jag1-Notch1* juxtacrine signaling between ECs and neurons and that this improves neuron activity as reflected by Ca^2+^ oscillations. In addition, delta-notch signaling in ECs may also improve the autocrine secretion of VEGF, which improves neurite outgrowth and fosters neuroprotection.

Experiments were also conducted with the neurospheres in the same gel region (contact culture) or in an adjacent gel region (non-contact culture) to assess the influence of direct juxtacrine interactions. These conditions proved to influence endothelial network formation as characterized by area coverage, the effective diameter of vessels and branch length, but not branch number (Supplementary Fig. S3). These characteristic differences were dependent on seeding density and concentration of the collagen gel as well as the concentration of growth factors such as VEGF^44^. Others have shown that co-culture with fibroblasts and mesenchymal stem cells^45^ improved vascular formation and stabilization by vasculogenesis through direct paracrine signaling, resulting in increased branch length, area coverage and effective diameter^46^. Our results are consistent with these previous observations^17,44^. In addition, our results indicate that co-culture also affects vascular formation via juxtacrine and paracrine signaling, for example via *delta-notch* (Fig. 4i). This might lead to increasing VEGF secretion which has been shown to decrease vascular morphological properties such as branch length, area coverage, and effective diameter (Supplementary Fig. S2)^44^.

### Co-culture of MN spheroids and iPS-derived ECs in a microfluidic device

**Figure 5:**
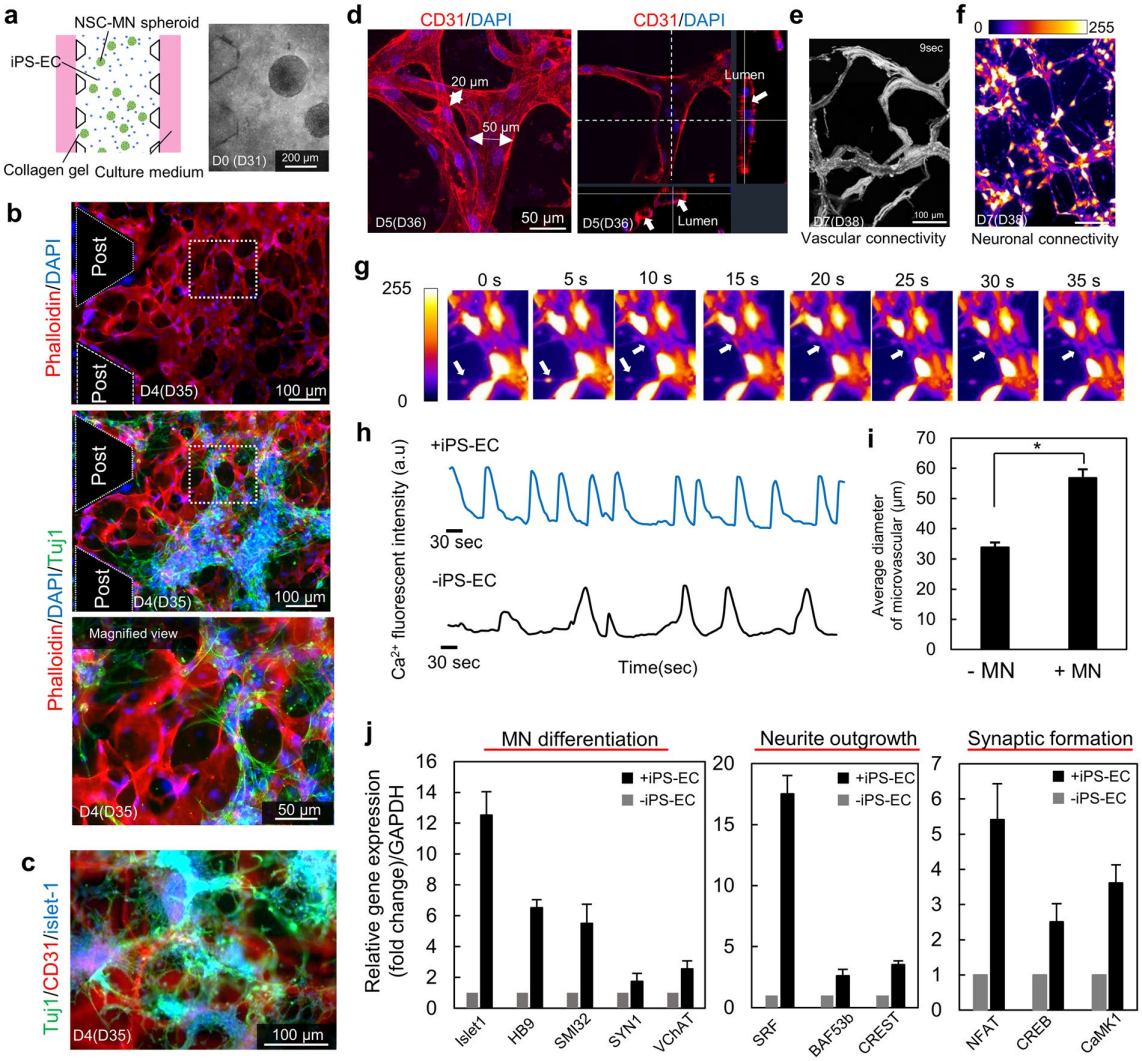
hiPS-vascular and hES-neuronal networks in a microfluidic device. (**a**,**b**,**c**) Formation of iPS-EC networks in a microfluidic device with MN spheroids. Microvascular networks were formed in 2–3 days. After formation of the microvascular network, MN spheroids start to extend protrusions and form neuronal networks intermingling with the microvascular networks. Magnified region is indicated in the white dotted box. (**d**,**i**) Lumen formation of iPS-EC vascular networks. The average diameter of networks is approximately 55 μm with MN networks, while the diameter is less than 40 μm without MN networks. n = 6; *P < 0.01, student’s t-test. (**e**) Evaluation of vascular connectivity by perfusion of 1 μm microbeads through microvascular networks. Images show the path lines of microbeads over a 9 second period. (**f**) Evaluation of neuronal connectivity by Ca^2+^ imaging stained by fluo-8-AM. Frequent Ca^2+^ oscillation can be observed in microfluidic devices. (**g**,**h**) Quantification of Ca^2+^ oscillation with and without iPS-EC networks. Local Ca^2+^ transportation was analyzed by post-image processing. (**g**) Frequency and amplitude of Ca^2+^ oscillation increased in the presence of iPS-EC networks. (**j**) Relative gene expression change between with and without EC networks after 7 days of static culture indicated that iPS-EC microvascular networks clearly upregulated the gene related to MN differentiation, neurite outgrowth, and synaptic formation. n = 3.

To overcome potential issues associated with using HUVECs (such as high permeability^16^ and non-physiological expression of transporters^47^), we use iPS-derived ECs in a multichannel microfluidic device (Fig. 1e). This has the combined advantages of culturing the cells in a confined space to enhance paracrine signaling, enabling perfusion of the vascular networks, and facilitating high-throughput drug screening. The microfluidic devices have 3D hydrogel scaffolds in multiple gel channels, microchannels for providing fresh medium and formation of an EC monolayer, and facilitate high-resolution imaging via a cover glass at the bottom of the device (Fig. 1c). Collagen gel (8–10 μl at 2.4 mg/ml) with iPS-ECs at a density of 3.0 × 10^6^ cells/ml and several MN neurospheres (diameter <150 μm) was injected into the gel channels (Fig. 5a). After crosslinking for 10 min, the culture medium was sequentially injected into each culture medium channel. EC networks formed in 1–2 days in the presence or absence of MN spheroids (Supplementary Fig. S2), and MNs started to migrate from the spheroids and extend neurites, resulting in the formation of interpenetrating vascular and neuronal networks in 4–5 days (Fig. 5b,c). The vascular networks formed lumens with an average diameter of ~60 μm (Fig. 5d,i, Supplementary Movie. S2).

The functionality of the vascular networks was evaluated in terms of the perfusability of the entire vascular system. Before this, iPS-ECs were seeded into the microchannel in order to form a monolayer in the media channel and to facilitate EC openings from the networks^17^. After 7 days of culture, perfusability was demonstrated by flowing 1 μm fluorescent beads into the culture medium channels, and acquiring time-lapse images at 20 frames/second (Supplementary Movie. S1 and Fig. 5e). Neuronal connectivity was demonstrated by Ca^2+^ imaging using Fluo-8AM to detect spontaneous and synchronized Ca^2+^ oscillation (Fig. 5f,g). Ca^2+^ oscillation was observed in many parts of the neuronal networks, and both the frequency and amplitude of Ca^2+^ oscillation increased in the presence of iPS-EC networks compared to experiments without iPS-EC networks (Fig. 5h). This supports the results in the macro co-culture of HUVECs and MN spheroids, in which paracrine and juxtacrine signaling with ECs were found to improve neuronal differentiation and function. Furthermore, gene expression with and without iPS-EC networks after 7 days of static culture which are related to MN differentiation (*Islet1*, *HB9*, *SMI-32*, *Synapsin1*, and *vChAT*), neurite elongation (serum response factor (*SRF*), *BAF53b*, and calcium-responsive transactivator (*CREST*)), and synapse formation (Nuclear factor of activated T-cells (*NFAT*)^48^, cAMP response element-binding protein 5 (*CREB*)^49^, and *CaMK1* were measured by RT-PCR (Fig. 5j). Hence, improvement of neural activity coincided with enhanced MN differentiation and synaptogenesis along with the mature synapse formation judging from the increase of *NFAT*, *CREB*, and *CaMK1*^50^.

### Effect of microvascular network perfusion on neural activity

**Figure 6:**
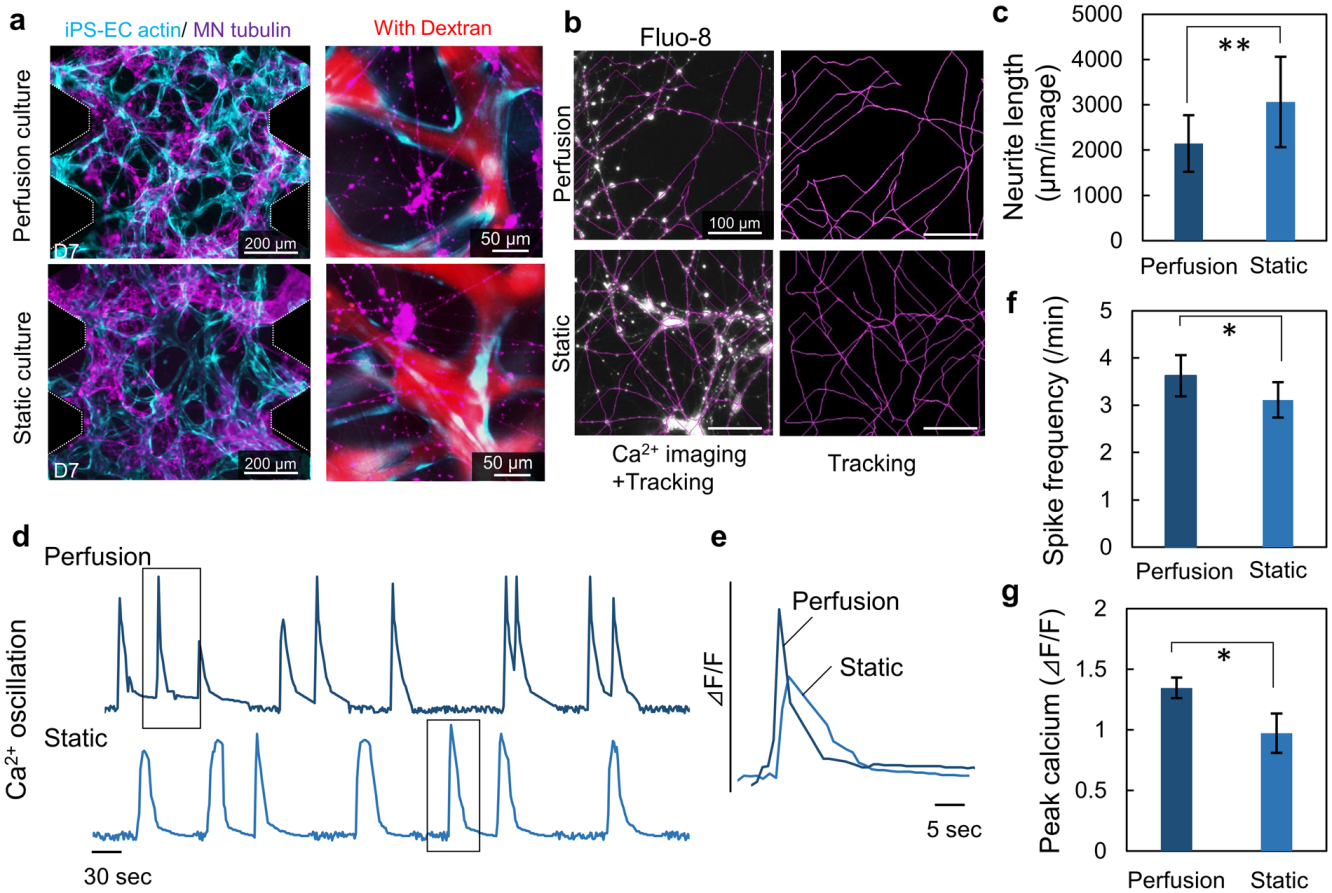
Perfusion culture influences synaptogenesis of MN networks along with synapse elimination. (**a**) Formation of hiPS-EC networks (cyan) and MN networks (purple) in a microfluidic device in the presence and absence of perfusion culture after 7 days of culture. There is no significant difference of formation of microvascular networks. Dextran perfusion demonstrated patent endothelial networks in both conditions. (**b**,**c**) Ca^2+^ imaging demonstrated that perfusion culture suppresses neurite extension compared to static culture quantified by neurite tracking (purple). (**d**) Spontaneous Ca^2+^ oscillation showed perfusion culture upregulated neural activity and synaptic connections, whereas total neurite length is shorter than static culture on day 7. (**e**) The representative waveform of Ca^2+^ imaging indicated that MN networks with perfusion culture higher response of depolarization of action potential than static culture. (**f**,**g**) Quantification of spike frequency and peak calcium concentration showed perfusion culture significantly accelerated synaptogenesis along with synapse elimination. n = 3; *P < 0.01, **P < 0.05, student’s t-test. Error bar ± SD.

To evaluate if microvascular network perfusion affects neural activity as well as vascular formation, the microvascular networks were subjected to intraluminal flow in the microfluidic system. The flow of culture medium was generated using a hydrostatic pressure head of 45 Pa. Large medium reservoirs were connected to the microfluidic devices to maintain a stable hydrostatic pressure (15% drop in 24 h). In both perfusion and static cultures, microvascular networks of iPS-EC and MN networks were established (Fig. 6a). There was no significant difference in vascular network morphology observed between perfusion and static culture (Supplementary Fig. S4). Furthermore, fluorescent-tagged 40 kDa dextran was perfused into the microvascular networks in order to visualize the perfusability and measure barrier function of the networks (Supplementary Fig. S5). Quantitative comparison using image analysis revealed that the total neurite length of MN networks with perfusion is less than in static culture at day 7 (Fig. 6b,c). On the other hand, neural activity characterized by Ca^2+^ imaging was enhanced in the perfusion culture. The frequency of neuron firing increased along with a concomitant increase in the concentration of BDNF (Fig. 6d,e,f, Supplementary Fig. S6). Interestingly, the pulse shape of Ca^2+^ indicator changed significantly with perfusion (Fig. 6e). This shape change with a sharper peak indicates that depolarization and hyperpolarization events occur more quickly, a further indication of neuronal network maturity^51^. Furthermore, peak calcium concentration in the presence of perfusion culture is higher than under static conditions (Fig. 6g).

## Discussion

Neuro-vascular coupling is important both to maintain neural function and activity and to form microvascular networks. This co-culture model with MN and microvascular networks in a microfluidic device facilitates investigation of paracrine interactions between these two types of networks as well as the effect of microvascular network perfusion. Spheroid culture NSC-MN and MNP-MN spheroids clearly improves differentiation into mature MNs (Fig. 2c,d) compared to 2D cultures. In addition, NSC-MN spheroids are composed of not only MNs but also contain more glial cells compared to MNP-MN spheroids (Fig. 2b), resulting in improved neurite elongation (Fig. 3f). This heterogeneity could potentially represent a more realistic *in vitro* model that could help in furthering our understanding of the pathology and pathogenesis of MND along with neurodegeneration. It is well known that glial cells accelerate the differentiation of MNs and regulate synapse formation^52–54^. On the other hand, astrocytes from ALS patients are toxic to MNs^55^; MNs are selectively sensitive to the toxic and non-cell autonomous effects of glial cells mediated by inflammatory factors such as interferon gamma (IFN-γ) and activating eNOS^56,57^. Further experiments are needed to study MN-glial interactions, but the novel use of NSC-MN spheroids in our model could be useful to test their interaction, or as a neurodegeneration model because they might capture the key role of astrocytes in the pathogenesis of MND.

Co-culture with ECs was found to improve neural activity as measured by Ca^2+^ imaging in macro (Fig. 3) and microfluidic cultures (Fig. 4). To understand the mechanistic basis of this, we focused on BDNF secretion by ECs, which stimulates neural activity and neural differentiation via the *MAPK/ERK* and *PI3K/Akt* pathways^40^ (Fig. 3g–i). In co-culture, BDNF secreted by ECs and juxtacrine signaling via *Dll4-Notch1* and *Jag1-Notch1* upregulates neural activity and MN differentiation (measured by increased gene expression of mature MN markers), neurite outgrowth, and synapse formation (Figs 3f and 4h,j).

In this work, we focused on cell-cell interactions via autocrine signals such as BDNF and juxtacrine signals via *Dll4-Notch1* and *Jag1-Notch1*. In addition to these interactions, there are other interactions between MN and vascular networks. For instance, a direct connection via ephrin B2–Eph singling between capillaries and NSCs has an important role in maintaining homeostasis and stemness, which prevents depletion of the stem cell pool^58^. These signals activate the corresponding receptors on NSCs that suppress proliferation and differentiation. As neural stem cells detach from the vascular networks, they start to proliferate and differentiate^59^ because Eph and Notch signaling are suspended. In addition, VEGF-C/VEGFR-3 signaling between ECs and NSCs, which is required for lymphangiogenesis, regulates adult neurogenesis^60^. It should be noted that this microfluidic culture platform also facilitates decoupling of paracrine and juxtacrine signaling by co-culturing in different gel regions adjacent to each other (Supplementary Fig. S3). Such a setup could potentially be used for investigating in greater detail the signaling pathways between ECs and NSC and other neurons in neural development, adult neurogenesis, and the pathogenesis of neurodegenerative disorders.

In addition to paracrine and juxtacrine signaling by ECs, capillary networks also improve the supply of oxygen and nutrients. The mechanical cues provided by flow through these networks likely plays a critical role in the maintenance of vascular networks as well as neural activity. In our model, perfusion culture improves robust synaptic formation and maturation but reduces neurite outgrowth (Fig. 6). Neural circuit formation involves several steps; migration, neurite elongation, axon guidance, synaptogenesis^61^. In particular, synaptogenesis is divided into critical steps such as synapse formation, synapse maturation, and synapse elimination. After synapse maturation, extra synapses are selectively eliminated along with regression of neurites. Perfusion of vascular and MN networks leads to synapse formation as well as synapse elimination, judging from Ca^2+^ imaging and measurement of total neurite length. The shorter neurites in the perfusion culture (Supplementary Fig. S7 and 6c) could be due to two possible factors. Either perfusion lowers the local concentration of growth factors or perfusion improves neuronal maturation leading to regression of immature neurites. Our observation of neurite maturation reflected by the Ca^2+^ signaling indicates improvement in neuronal maturation. Based on this, we lean more towards the second hypothesis. The lower neurite length and the increased MN activity could also be affected by the shear stress. The shear stress generated by perfusion of the microvascular networks upregulates eNOS synthesis in ECs^62,63^ subsequently leading to increased expression of mature BDNF^64,65^. Mature BDNF then stabilizes the synapse along with synaptic maturation and elimination via TrkB pathway^66,67^. In further probing this phenomenon, we found that BDNF concentration increased in the presence of perfusion culture (Supplementary Fig. S5). In addition, permeability measurements showed that microvascular networks created by iPS-EC networks exhibit very low permeability values and that co-culture with MNs reduces permeability even further (Supplementary Fig. S5). Perfusion, on the other hand, did not influence permeability. A recent study showed that human iPS derived ECs could be differentiated into brain-specific ECs by co-culture with neurons and astrocyte^68^. Our model could be adapted to use these brain ECs to form microvascular networks to tailor the model even more towards brain disorders. Further investigation is needed to fully validate the biological relevance of our model and how it represents the *in vivo* cross-talk between EC networks and MN networks. There is room for improvement in terms of using more organ-specific cells or brain organoids^69^ as well as disease specific patient derived cells. However, the culture platform as is, with both EC and MN networks in a microfluidic device facilities such investigations and could be as a drug screening model for motor neuron disease.

## Conclusion

This study demonstrates the simultaneous formation of 3D interpenetrating vascular and MN networks in macro and microfluidic systems. The formation of human NSC-MN spheroids that contain both MNs and glial cells promoted rapid differentiation and exhibited long-term stabilization. Co-culture with ECs improved neurite elongation and robust neuronal activity. This improvement may be caused by paracrine signaling from ECs mediated by BDNF, and direct juxtacrine singling via the *delta-notch* pathway. Furthermore, these microvascular networks with neuronal networks could be perfused when grown in a microfluidic system as tested by the perfusion of fluorescent beads. Interestingly, not only did ECs benefit neuronal differentiation, but vascular formation also benefitted from the presence of neuronal networks through paracrine signaling. The microfluidic system experiments enable us to investigate perfusability of the microvascular networks and offers the potential to study the effect of mechanical stimuli such as shear stress. We found that perfusion culture also improves MN activity along with mature synaptogenesis. This 3D *in vitro* model of vascular and neuronal networks could be useful for understanding the pathogenesis of MND and for drug screening. It also has broader implications for tissue engineering and the study of neural development.

## Materials and Methods

### Cell culture

Human umbilical vein ECs (HUVEC, Lonza) were sub-cultured with endothelial growth medium (EGM-2). Passage 4 HUVECs were used in all vasculogenesis experiments. iPS-derived ECs (iCell Endothelial cell, Cellular Dynamics International) were cultured on a fibronectin-coated petri dish (50 μg/ml) with iCell endothelial growth medium supplemented with VascuLife VEGF medium complete kit including rhVEGF, rhEGF, rhFGF, rhIGF-1, Ascorbic acid, hydrocortisone hemisuccinate, heparin sulfate and L-glutamine. iPS-EC of less than passage 5 were used in all vasculogenesis experiments. H9 human ES-derived neuronal stem cells were cultured on a coating of CTS CELLstart with DMEM/F-12 medium supplemented with 2 mM GlutaMAX-I supplement and 20 ng/ml bFGF, 20 ng/ml EGF and 2% StemPro Neuronal Supplement (all from Gibco). For differentiation into MNP cells via caudalization and ventralization, hNSC were subcultured on ornithine-laminin coated dishes and the medium was replaced with StemPro hESC SFM (Gibco) supplemented with SHH (200 ng/mL), RA (50 μM), bFGF (8 ng/mL) and Activin (10 ng/mL). In the process of MN maturation, the medium is replaced with StemPro hESC SFM medium supplemented with 10 ng/mL of BDNF and GDNF. All cells were cultured at 37 °C with 5% CO_2_ in a humidified incubator.

To clarify our choice of cells, it would have been appropriate to use iPS-ECs for all cultures rather than HUVECs due to their tendency to become organ specific. However, we did not use iPS-ECs in macro-cultures and used HUVECs instead because of the limited iPS-EC availability. For macro-cultures, we need many more iPS-ECs cells (by an order of magnitude) to create microvascular networks with the MN spheroids (we need at least 50 times as many cells compared to a microfluidic device). So, it is difficult to expand the cells maintaining a low passage number (iPS-EC proliferate slowly compared to HUVECs) and for forming good microvascular networks a low passage number is necessary which is not possible with iPS-ECs. In contrast, in a microfluidic system, far fewer iPS-ECs are needed, even when preparing many biological replicates, due to the small gel volumes.

### Microfluidic device fabrication

Microfluidic devices made of polydimethylsiloxane (PDMS) silicone elastomer kit (Dow Corning, sylgard184) were fabricated by soft lithography from SU-8 patterned wafers. Silicone elastomer and a curing agent mixed at a weight ratio of 10:1 was degassed, poured onto the photolithographically patterned SU-8 structures and cured in the oven at 80 °C for 6 h. Inlet and outlet ports were created with biopsy punches (1 mm and 3 mm) and the devices were sterilized by autoclave. Glass coverslips were plasma bonded to the PDMS layer to create channels ~250 μm in height. The multichannel of the hydrogel are 1.25 mm wide, while the culture medium channels are 1.7 mm wide. Before injection of the hydrogel, the microchannels were coated with poly D lysine (PDL) for 30 min.

### Motor neuron spheroid formation

Neurospheroids were created using a spindle-shaped bottom 96 well plate (PrimSurface 96 M) and PDMS based spheroid array chips. As a negative mold, an array of microwell (diameter 500 μm, depth: 500 μm) was fabricated in an olefin plate using a computer-controlled drilling machine (Shopbot Desktop). To transfer the structures of the negative mold to a positive mold, a mixture of epoxy resin (epoxy: curing agent, 2:1) was poured into the negative mold and cured for 72 h at room temperature. PDMS solution (prepolymer solution: curing agent, 10:1) was poured into a positive mold and cured in an oven at 60 °C for at least 12 h. After autoclave (120 °C for 1 h), the chip was treated with 1 ml of 4% Pluronic F-127 in PBS for 6 h to reduce cell attachment to the PDMS surface.

Neural stem cell-derived MN (NSC-MN) spheroids were formed in 24 h by seeding hNSC in the spheroid formation plate. Then, the culture medium was replaced with differentiation medium supplemented with retinoic acid (RA), sonic hedgehog (SHH), bFGF and activin A for cell fate determination via caudalization and ventralization. After additional culture of 18 days, the culture medium was replaced again, supplemented with RA, brain-derived neurotrophic factors (BDNF) and glial cell-derived neurotrophic factor (GDNF) for maturation of MNs and cultured for 7 more days. Then, the culture medium was switched to medium without RA for an additional 7 days. To create motor neuron progenitor (MNPs) cell-derived MN (MNP-MN) spheroids, at first, hNSCs were differentiated into MNPs in a Petri dish with differentiation medium supplemented with RA, SHH, bFGF and Activin A. After caudalization and ventralization (typically after 18 days of differentiation), MNP cells were seeded onto a spheroid formation plate. Then, MNP-MN spheroids were matured using the same protocol. By changing the seeding density of cells in the spheroid formation plate (3.0 × 10^3^ cells/well to 2.5 × 10^4^ cells/well), spheroids with different diameter were obtained (Fig. 2a). To consider necrosis inside the core of the spheroid, spheroids with diameter <200 μm were used in subsequent experiments. To evaluate neurite elongation potential, neurospheres were embedded in rat tail collagen gel (BD falcon).

### Co-culture of MN spheroids and ECs

To evaluate the interaction between MN spheroids and ECs, they were simply co-cultured in rat tail collagen gel (for HUVEC) or porcine skin collagen gel (for iPS-EC). ECs were embedded at a density of 3.0 × 10^6^ cells/ml and neurospheres were embedded at a density of approximately 10 spheroids in 500 μl of collagen gel on a Petri dish. At the beginning of culture, EGM-2 and iPS-EC medium were used for co-culture. After 24 h, the culture medium was replaced with 1:1 mixture of EGM-2 or iPS-EC and StemPro hESC SFM medium (final concentration of BDGF and GDNF, 10 ng/ml). In the microfluidic device, 10 MN spheroids suspended in a porcine skin collagen gel (Nitta Gelatin) were injected into gel channels with iPS-ECs. For perfusion culture, a hydrostatic head (45 Pa) was generated between the two media channels 24 h after cell seeding. To maintain stable pressure, the large medium reservoir (upstream) was connected to the outlet of the microfluidic device and the hydrostatic head was reset every day by adding perfused culture medium (collected from the downstream reservoir and added to the upstream one). All medium was replaced with fresh culture medium every 2 days.

### Immunostaining

Cells in petri dish and microfluidic device were fixed with 2% paraformaldehyde in culture medium for 15 min in the incubator then, replaced with 4% paraformaldehyde in PBS to preserve neuronal structures. They were then treated with 0.1% Triton X 100 for 10 min, and stained with rhodamine phalloidin and actin-stain 488 phalloidin (Cytoskeleton, Inc., Denver, CO, USA) and DAPI for 20 min at room temperature. For immunostaining of Tuj1, VE-cadherin, HB9 and islet1, cells were incubated with rabbit anti-human VE-cadherin (1:200, Biolegend), mouse anti-neuron-specific βIII tubulin (Tuj1 1:100, Abcam), rabbit anti-human HB9 (1:200, Abcam) and rabbit anti-human islet1 (1:100, Abcam) for 1 h at room temperature, incubated with secondary antibodies of Alexa Fluor 555 anti-rabbit IgG (H + L) (1:500), Alexa Fluor 405 anti-rabbit IgG(H + L) (1:500), Alexa Fluor 488 goat anti-mouse IgG (H + L) (1:500), Alexa Fluor 488 goat anti-rabbit IgG (H + L) (1:500) for 1 h at room temperature, followed by three washes in PBS. The neurite elongation length and vasculogenesis of ECs were analysed from the reconstructed three-dimensional images with image analysis software (IMARIS, Bitplane, Switzerland). Cells were observed using a fluorescence microscope (Axiovert 200, Zeiss, Germany) and a confocal laser scanning microscope (FV-1000, Olympus, Japan).

### Real-time PCR analysis

To separate ECs and MNs from microfluidic devices, the collagen gel was dissolved by collagenase and the cells were sorted by FACS (BD FACSAria III cell Sorter, EC marker: CD31 or neural marker: MAP2). Total RNA was isolated from cells with the RNeasy mini kit (Qiagen). Reverse transcription was performed using a SuperScript cDNA kit (Invitrogen). Primer sequence is given in Table S1. Real-time PCR was performed with an Applied Biosystems 7900HT Fast Real-Time PCR System, using a Power SYBR Green Master kit. Glyceraldehyde 3-phosphate dehydrogenase (GAPDH) mRNA as housekeeping gene set to 100% was used as the internal standard in all experiments. The RT-PCR experiment was repeated at least 3 times for cDNA prepared from three batches. For the data in Fig. 2b, the day 14 gene expression results for the MNP-MN culture is obtained from the monolayer stage (as illustrated in Fig. 1f) because we form MNP-MN spheroids only from day 18.

### ELISA analysis

Culture medium was collected every 2 days and stored −20 °C to measure growth factor concentrations. BMP-2 and BDNF were measured by ELISA (BMP-2 human ELISA kit, Thermo Fisher Scientific and Human Free BDNF Quantikine ELISA kit, R&D systems); a microplate reader was used to measure the absorption of each sample at 495 nm. The amount of BDNF and BMP-2 was calculated using a calibration curve based on the known concentration. This experiment was carried out in triplicate.

### Ca^2+^ oscillation imaging

To measure Ca^2+^ oscillation, cells were washed with PBS (without Ca^2+^, Mg^2+^) at three times. Then, Fluo8-AM (5 μM) with recording medium (20 mM HEPES, 115 mM NaCl, 5.4 mM KCl, 0.8 mM MgCl_2_, 1.8 mM, CaCl_2_, 13.8 mM glucose) was added and incubated for 1 h at 37 ^**°**^C. After loading Fluo8-AM, it was replaced with the recording medium. Time-lapse movies were acquired for 10 min (resolution: 680 × 512, exposure time: 10 msec) using a fluorescent microscope (Axiovert 200, Zeiss).

### Perfusability of vascular networks

After formation of vascular networks in a microfluidic device, iPS-ECs were seeded into the medium channel at a density of 5.0 × 10^6^ cells/ml to create openings for the lumen structures into the media channel. Then, 1.0 µm fluorescent beads (FluoSpheres Carboxylate-Modified Microspheres) in culture medium were perfused into the microvascular networks using a syringe pump (50 µL/min). The movie was acquired for 10 seconds (20 fps) using a fluorescent microscope (Axiovert 200, Zeiss).

### Quantification of vessel permeability coefficient

To determine the permeability coefficient, 40 kDa fluorescent tracers (Texas red dextran) was introduced through the microvascular networks by imposing a hydrostatic pressure drop across the gel region between the two medium channels. Vascular permeability is computed as the flux of solute across the walls of the vascular network divided by the concentration difference. Using mass conservation, the quantity of Texas red-dextran crossing the vascular network equals the rate at which it accumulates outside the vessels in the tissue gel region. The formula to calculate permeability coefficient was previously described^70^. The fluorescence intensity values, vessel surface area and tissue/gel region area were computed using ImageJ.

### Statistical and image analysis

The neurite morphological profiles in 3D culture were quantified using Imaris and ImageJ. First, the neurite structures were identified from fluorescent images stained with Tuj1 using the plugin of filament tracer with spine in the Imaris software, then the rendering images were saved as TIF files. The TIF files were then loaded in Image J and neurite length and spine density were quantified by the Image J plugin, Neuron J. Fluctuation amplitudes of the Ca^2+^ indicator were also quantified by ImageJ. Time-lapse images captured by fluorescence microscopy were converted to grayscale (16 bit) and ROIs were calculated by the change of fluorescent intensity of Fluo-8 in circular regions in cells using ImageJ. Based on these ROI values, graphs of intensity changes were generated. To quantify the vascular structure in the microfluidic device, 3D images captured by confocal microscopy were converted to maximum projection intensity images and binarized. Then, area coverage, effective diameter, branch number and branch length of the microvascular networks were quantified using the ImageJ plugin AnalyzeSkeleton (http://fiji.sc/AnalyzeSkeleton). The data are expressed as mean values ± SD, and one-way or two-way ANOVA tests were performed. If significant, the data were tested with multiple comparison tests (Tukey-Kramer post hoc tests, n = 10; **p < 0.05, *p < 0.01). The tests were performed using MATLAB software (Math Works, Worcester, MA).

## Electronic supplementary material

Supplementary data

Supplementary Movie S1 Perfusion of microbeads through microvascular network of iPS-EC and neuronal networks on day 7

Supplementary Movie S2 3D rendering of vascular networks

Supplementary Movie S3 Ca2+ imaging of neuronal networks in microfluidic device
