# Supplementary material for: Engineered 3D vascular and neuronal networks in a microfluidic platform
Source: Sci Rep. 2018 Mar 26;8:5168. doi: 10.1038/s41598-018-23512-1 (PMC5979969; doi:10.1038/s41598-018-23512-1)
Supplement: Supplementary file 1 — Supplementary data [file 41598_2018_23512_MOESM1_ESM.docx]

##

## Supplementary data

## Engineered 3D vascular and neuronal networks in a microfluidic platform

Tatsuya Osaki^1,^ Vivek Sivathanu^1^ and Roger D. Kamm^1, 2, 3^

^1^ Department of Mechanical Engineering, Massachusetts institutes of Technology, 77 Massachusetts Avenue, Cambridge, MA, 02139, USA.

^2^ Department of Biological Engineering, Massachusetts institutes of Technology, 77 Massachusetts Avenue, Cambridge, MA, 02139, USA.

^3^ Singapore-MIT Alliance for Research & Technology, Singapore, Singapore.

* Corresponding author

E-mail: rdkamm@mit.edu

S**upplementary Figure S1 | Representative images on day 18 to quantify spreading area, neurite length and spine density.**

NSC-MN spheroid (left) and MNP-MN spheroid on laminin coated dish 18 days after reseeding.

**Supplementary Figure S2 | iPS-EC networks without neuronal networks in a microfluidic device.**

(A) iPS-EC microvascular networks in a microfluidic device on day 4 without MN networks (B) VE-cadherin staining show that these are functional networks as evidenced by adherence junctions and formed luminal structures.


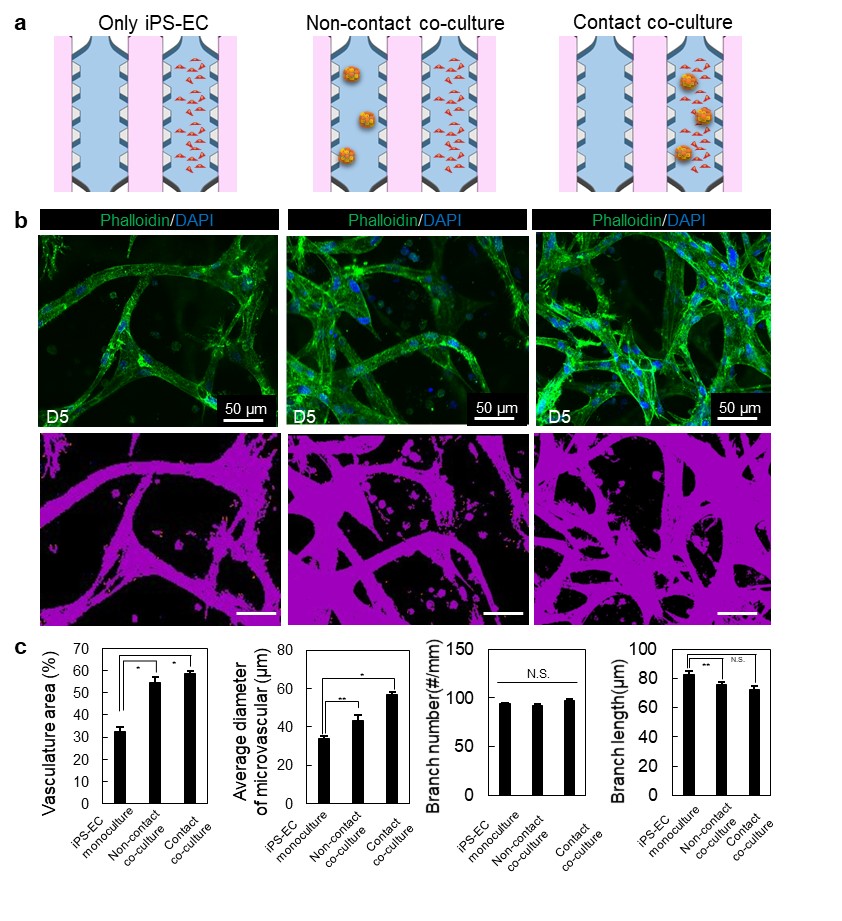


**Supplementary Figure S3| Characterization of microvascular networks under three different conditions (iPS-EC monoculture, non-contact co-culture, contact co-culture with iPS-EC and MN spheroids).**

(a) Microvascular networks by staining Phalloidin (Green) and DAPI (blue) of only iPS-EC and iPS-EC and MN spheroids in different gel channels, and the same gel channel. (b) Images were binarized and converted to skeletal images to calculate area coverage of vasculature, average diameter, branch number and branch length (c).

**
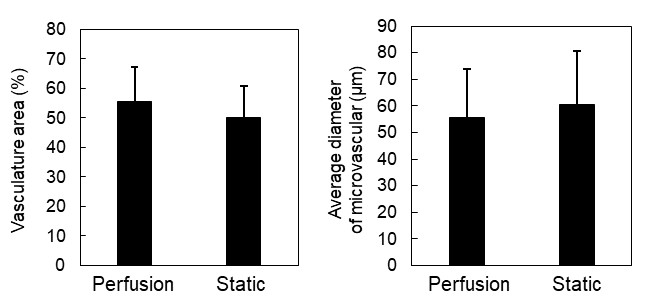
**

**Supplementary Figure S4| Vascular area and diameter of microvascular networks in perfusion and static culture**

No significant differences of vasculogenesis in microfluidic device between perfusion and static culture in terms of vasculature area and diameter of microvascular networks.

**Supplementary Figure S5| Permeability of iPS-EC microvascular networks with and without perfusion**

Permeability coefficient was measured by flow Texas-red tagged 40 kDa dextran solution. No significant difference between static and perfusion culture. MN networks help low permeability of iPS-EC microvascular networks.

**
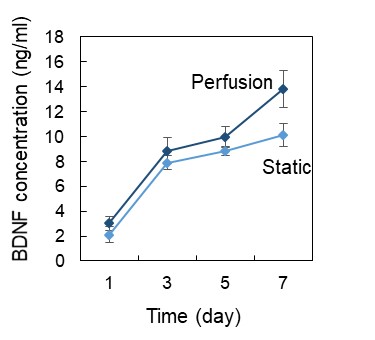
**

**Supplementary Figure S6| Perfusion culture upregulate BDNF productions secreted by motor neuron cells.**

Culture medium was collected from microfluidic device every 2 days. BDNF concentration was detected by ELISA measurement. BDNF concentration in perfusion culture condition is higher than static condition over time.


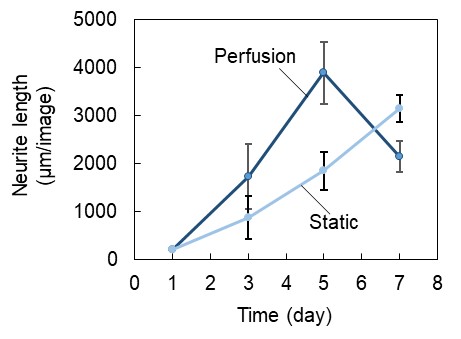


**Supplementary Figure S7| The neurite lengths in perfusion and static culture in the microfluidic device**

Neurite lengths in perfusion and static condition were quantified by tubulin staining using ImageJ every 2 days.

**Supplementary Table S1.** Primer information for real-time RT-PCR

| Target gene | Forward primer sequence(5’-3’) | Reverse primer sequence(5’-3’) |
| --- | --- | --- |
| GAPDH | ACC ACA GTC CAT GCC ATC AC | TCC ACC ACC CTG TTG CTG TA |
| Nanog | CCT GTG ATT TGT GGG CCT G | GAC AGT CTC CGT GTG AGG CAT |
| Nestin | GTC TCA GGA CAG TGC TGA GCC TTC | TCC CCT GAG GAC CAG GAG TCT C |
| OLIG2 | CCT GAG GCT TTT CGG AGC | CTG GCG TCC GAG TCC AT |
| HB9 | CTT TTT GCT GCG TTT CCA TT | GCA CCA GTT CAA GCT CAA CA |
| Islet1 | CAT GCT TTG TTA GGG ATG GG | ACG CAT CAC GAA GTC GTT C |
| HIF1-alpha | CAG CTT CCT TCG GAC ACA TAA G | CCA CAG CAA TGA AAC CCT CCA |
| Hes1 | CGA AAA TGC CAG CTG ATA TAA | ACA CGC TCG GGT CTG TGC TGA |
| Hes5 | CTC CGC TCC GCT CCG CTC GCT AAT | GGG GCC GCT GGA AGT GGT AAA GCA |
| Notch1 | ATG CAG AAC AAC AGG GAG GA | TAT GAT CCG TGA TGT CCC GG |
| Notch4 | TCA GCC ACC AGT GTC AGA AT | GGG TCT CAC ACT CAT CCA CA |
| Delta-like1 | CAG AAA GAC TCA TCA GCC GC | CAG CCC TCT CCG TAG TAG TG |
| Delta-like4 | CGC TAC TCT TAC CGG GTC AT | ATC TGG CTG GCA CAC ATA GT |
| Jagged1 | CCT GAA GGG GTG CGG TAT AT | CAT GGC AGT ATG TTC CCG TG |
| Jagged2 | CAA CCC CTG TGT GAA TGG TG | ATT GTA GCA AGG CAG AGG GT |
| Neurogenin-2 | TAC CTC CTC TTC CTC CTT CA | GAC ATT CCC GGA CAC ACA C |
| SMI-32 | GCA GTC CGA GGA GTG GTT C | CGC ATA GCG TCT GTG TTC A |
| Synapsin I | AGT TCT TCG GAA TGG GGT GAA | CAA ACT GCG GTA GTC TCC GTT |
| VAChT | CTG CTA GTG AAC CCC TTG AGC | CAG GAC TGT AGA GGC GAA CAT |
| SRF | CGA GAT GGA GAT CGG TAT GGT | GGG TCT TCT TAC CCG GCT TG |
| BAF53b | GCA CTA CGA GAT GCC CAA TG | TTG ACG TTC GAG GGA TCA AAC |
| CREST | CAA GGC ACC ATC GGC AAC TA | CTG CTG CAT CAT GGA GAC TG |
| NFAT | GCT GGA TAA CAG TCG GAT GTC | GCC TCT GCT TTG GAT TTC GTT |
| CREB | CCC TGC CCA ACC CTA CAA TG | GGA CCT TGC ATC CCC ATG AT |
| CaMK1 | TGC TGT CCT GCA CAA GAT CAA | GCT CCG TGT AGA AGC CTT TTT |

**Supplementary Movie S1 Perfusion of microbeads through microvascular network of iPS-EC and neuronal networks on day 7**

**Supplementary Movie S2 3D rendering of vascular networks**

**Supplementary Movie S3 Ca^2+^ imaging of neuronal networks in microfluidic device**
